# Elements That Underpin the Design, Development, and Evaluation of Social Media Health Interventions: Protocol for a Scoping Review

**DOI:** 10.2196/31911

**Published:** 2022-02-01

**Authors:** Mohammed Zayan Nizam, Leigh Powell, Nabil Zary

**Affiliations:** 1 Institute for Excellence in Health Professions Education Mohammed Bin Rashid University of Medicine and Health Sciences Dubai United Arab Emirates; 2 School of Medicine Queen’s University Belfast Belfast United Kingdom

**Keywords:** social media, health intervention, behavior change models, health improvement, intervention design, models of design, evaluating interventions

## Abstract

**Background:**

Social media use has grown tremendously over the years. Given the volume and diversity of people on social media and the amount of information being exchanged, it is perhaps unsurprising that social media is being used as an avenue to disseminate and deliver health interventions. There exists an opportunity for social media health interventions to make a positive impact on health. However, there is a need to understand more about the ways in which these interventions are designed, developed, and evaluated. This scoping protocol will review the current state of this field by charting the elements that drive the design, development, and evaluation of these interventions. This includes charting models, frameworks, and rationales for the interventions, as well as the platforms being used, and the health behaviors being targeted. This intention of this scoping review is to help inform those who wish to develop effective social media health interventions.

**Objective:**

The objective of this review is to map the elements that drive the design, development, and evaluation of social media health interventions. We define “social media health intervention” as interventions that make use of social media platforms to disseminate or deliver health-related information and educational initiatives to the public. We will seek to chart the elements that drive the design, development, and delivery of such interventions, including their platforms and targeted health behaviors.

**Methods:**

The methodological framework for this review is guided by Arksey and O’Malley and enhancements by later studies. We will search relevant literature from 9 databases: (1) PubMed, (2) PsycINFO, (3) EMBASE, (4) Web of Science, (5) Scopus, (6) CINAHL, (7) ERIC, (8) MEDLINE, and (9) Google Scholar. The literature will be screened by at least two reviewers in 2 stages: (1) title/abstract screening against the eligibility criteria; and (2) eligible articles will then undergo a full-text screening. Data will be charted using the data charting tool developed by the authors.

**Results:**

The results of this study will be presented in a final scoping review paper, divided into 2 sections. The first section will describe the search strategy and study selection process and will contain the Preferred Reporting Items for Systematic Reviews and Meta-Analyses (PRISMA) flowchart. The second section will provide key details pertaining to the review objective and question.

**Conclusions:**

This review will help guide scholars looking to build social media health interventions toward evidence-based practices in design and evaluation.

**International Registered Report Identifier (IRRID):**

PRR1-10.2196/31911

## Introduction

### Background

Social media is inextricably linked to our lives. The use of social media has grown enormously over the years. As of January 2021, an estimated 4.6 billion people have access to the internet of which 4.2 billion people are social media users. Even taking into consideration multiple accounts and business accounts, a significant number of the population use and interact on social media [1]. Social media is defined as “forms of electronic communication (such as websites for social networking and microblogging) through which users create online communities to share information, ideas, personal messages, and other content” [2]. This definition may not necessarily capture the diversity of social media today. Still, the tenets of social media such as communication, user generation of content, and information sharing are seen across a host of social media platforms ranging from Facebook and Instagram to Twitter.

Social media users are diverse across ages, ethnicities, education levels, and come from a host of different backgrounds [1]. Having access to this diverse group of people creates a unique opportunity to use social media platforms as a means of promoting and disseminating health interventions. A health intervention is an act performed for, with, or on behalf of a person or population whose purpose is to assess, improve, maintain, promote, or modify health, functioning, or health conditions [3]. Social media platforms have been recognized and used for health interventions and hold tremendous potential to help elicit positive health behavior changes [4,5]. For example, health interventions to promote nutritional education have used social media platforms to identify behaviors and offer intervention [6]. Other social media health interventions include sexual health promotion [7], interventions for diabetes [8], and interventions targeting addiction and recovery [9].

The use of social media as a conduit for health communication is well established [10]. Social media provides health care professionals a platform to freely share information and combat health misinformation [11,12]. Social media health interventions also provide an opportunity to improve public health outcomes, such as in the recent COVID-19 pandemic where they have been used to disseminate information and increase vaccine uptake [10,13-16]. The rising use of social media and other digitized technology for health intervention has even led to the emergence of a new field called “Digital Public Health,” which is defined as “the use of technology, new types of data, and new ways of working that come with the digitization of public health and associated data’’[17].

To best leverage the opportunities and affordances of utilizing and disseminating health interventions over social media, there exists a responsibility to ensure that the design, development, and evaluation of such interventions are done in a systematic and evidence-based manner. There is a need to scope the current landscape of social media health interventions to better understand the elements which underpin these interventions. The last paper that took a broad look into the use of social media for health promotion and intervention was back in 2011, which primarily focused on the features of social media platforms [13]. Since then, the landscape of social media has changed, and the design of interventions has had to change with it. For those looking to build successful interventions, a need exists to scope the current landscape to understand what elements drive the design, development, and evaluation of social media health interventions.

### Objectives

The objective of this review is to map the elements that drive the design, development, and evaluation of social media health interventions. We define “social media health intervention” as interventions that make use of social media platforms to disseminate awareness or deliver health-related information and educational initiatives to the public. As such, only studies that describe a health intervention in which social media is the conduit for dissemination or delivery will be considered for review. Health interventions that do not use social media will be excluded. For those included papers we will seek to chart the elements that drive the design, development, and delivery of such interventions, including their platforms and targeted health behaviors.

## Methods

### Overview

This scoping review will be guided by the methodology proposed by Arksey and O’Malley [14] with updated enhancements proposed by Levac et al [18] and Peters et al [15]. This framework was chosen for its clarity in the stages of conducting a scoping review, including (1) identification of the research question; (2) identification of studies; (3) selection of studies; (4) charting the data; and (5) collating, summarizing, and reporting the results. The Preferred Reporting Items for Systematic Reviews and Meta-Analyses extension for Scoping Reviews (PRISMA-ScR) checklist will also provide further details and clarity [16].

### Stage 1: Identifying the Research Question

The primary question for our review is to understand the elements that drive the design, development, and evaluation of social media health interventions. The research questions below are aligned with our objectives of scoping the current landscape in order to inform those who wish to design evidence-based and effective social media health interventions. The following questions will guide our review:

- What are the underlying elements that inform the design, development, and evaluation of social media health interventions? What evidence-based models, frameworks, or theories underpin the design, development, and evaluation, if any?
- For what purposes are social media health interventions implemented? What behaviors are targeted, if any?
- What social media platforms are most pervasive in social media health interventions?
- What are the demographic characteristics of the populations targeted with social media health interventions?

The authors arrived at these questions after extensive discussions and brainstorming with the research team.

### Stage 2: Identifying Relevant Literature

A comprehensive review of literature will be conducted using the following databases : (1) PubMed, (2) PsycINFO, (3) EMBASE, (4) Web of Science, (5) Scopus, (6) CINAHL, (7) ERIC, (8) MEDLINE, and (9) Google Scholar. To capture relevant gray literature, we will conduct a search on Google Scholar. We will use an effort-bounded stopping criteria in which we examine the first 100 search results, then reassess, continuing to the next page of results, iteratively assessing if the results remain relevant to our scope [19].

Keyword search terms for this review were developed through discussion between the protocol authors. Input from a subject librarian was also sought to ensure keywords captured relevant data and the databases were appropriate. The keywords are based on the 2 overarching concepts of the review concept: social media and health intervention (Tables 1 and 2). A sample search strategy is provided in Table 3. Any amendments to the search strings will be thoroughly documented in the final scoping review.

**Table 1:** List of keywords used for the concept of “social media.”

Serial number	Keywords: Concept #1 “social media”
1	Social Media*
2	Social Network*
3	Social Platform
4	Twitter
5	Facebook
6	Instagram
7	Snapchat
8	TikTok
9	YouTube
10	WhatsApp
11	WeChat

**Table 2:** List of keywords used for the concept of “health intervention.”

Serial number	Keywords: Concept #2 “health intervention”
1	Health Promotion
2	Health Improvement
3	Health Maintenance
4	Therapeutic Intervention
5	Health Modification
6	Health Intervention

**Table 3:** Sample search strategy.

Search strategy	Database	Results
(“social media*”[Title/Abstract] OR “social network*”[Title/Abstract] OR “social platform”[Title/Abstract] OR “Twitter”[Title/Abstract] OR “Facebook”[Title/Abstract] OR “Instagram”[Title/Abstract] OR “Snapchat”[Title/Abstract] OR “TikTok”[Title/Abstract] OR “YouTube”[Title/Abstract] OR “WhatsApp”[Title/Abstract] OR “WeChat”[Title/Abstract]) AND (“health intervention”[Title/Abstract] OR “health intervention”[Title/Abstract] OR “Health Promotion”[Title/Abstract] OR “Health Improvement”[Title/Abstract] OR “Health Maintenance”[Title/Abstract] OR “Therapeutic Intervention”[Title/Abstract] OR “Health Modification”[Title/Abstract])	PubMed	1073

### Inclusion and Exclusion Criteria

As we wish to broadly map the existing literature, we will include a variety of studies, including published and gray literature. Studies to be reviewed will consist of those that use social media to disseminate or deliver a health intervention. Studies that describe a health intervention but do not use social media as a core component of dissemination or delivery will be excluded. A temporal limit will also be set, ensuring that only papers from 2005 to date will be reviewed. The date restriction has been set in consideration of the public release and advent of modern social media platforms [20]. Only studies in English will be reviewed. Textbox 1 summarizes our inclusion and exclusion criteria. These will be modified iteratively if needed.

**Summary of inclusion and exclusion criteria.**

*Inclusion criteria*

- Literature outlining health interventions that are delivered or utilized a publicly available social media platform.
- Studies that describe the design and implementation of the intervention or the evaluation of the intervention.
- Studies published within or after 2005.

*Exclusion criteria*

- Studies that describe health interventions but do not have any context of social media.
- Studies solely mentioning other uses of social media for health.
- Studies in languages other than English.

### Stage 3: Study Selection

The study selection for this review will follow a 2-step screening process. First, after databases and gray literature searches are concluded, results will be imported into a citation manager. Deduplication will be conducted at this stage, and all remaining studies will be imported to a review software called Rayyan [21]. Second, 2 reviewers will independently screen title–abstracts against the exclusion and inclusion criteria to determine which studies will be included in full-text screening. Interrater disagreement will be solved through discussion with a third independent mediator.

### Stage 4: Data Charting

Relevant data will be charted out from all included studies by 2 or more independent reviewers. The reviewers will develop and pilot a data extraction instrument that will be refined iteratively as we carry out the extraction process. Extracted data will include article details such as author/s, year of publication, evaluation method, design model, behavioral model, social media platform, audience type, and outcomes. A draft data extraction tool can be found in Table 4.

**Table 4:** Draft charting table.

Type of data	Details of charted data
Article information	Title Author/s Date of publication
What health behavior/condition was the target of the health intervention?	Health behavior/condition targeted
Social media platform used	Name of platform
How were participants recruited?	Recruitment method
What are the demographics of the population that the intervention was targeted at?	Age of users Country of user Language
What rationale was employed for the design of the intervention?	Models Frameworks Rationale Evidence based (yes/no)
Was the intervention evaluated? If so, describe the elements used to evaluate.	Models Frameworks Tools

### Stage 5: Collating, Summarizing, and Reporting the Results

We will present our charted data both graphically and narratively. Thoughtful consideration to using text and images to illustrate our results will be undertaken. In our summary will we consider the broader implications of our findings as we hope our review will provide practical context and guidance for those desiring to create social media health interventions.

### Ethics and Dissemination

Ethics approval was not required for this study. Future dissemination of the final review will include publication of the final scoping review in a peer-reviewed journal with a good impact factor.

## Results

The results from the extracted data will be presented in 2 broad sections, the first outlining the selection process and PRISMA flowchart. The second section will include results pertaining to our review question. The results will be presented in the final scoping paper.

## Discussion

Our scoping review will provide insights into the current landscape of health interventions that utilize social media. This study is not without limitations. Papers in languages other than English will not be included due to the limitations of the reviewers. Papers describing health interventions that do not use social media will be excluded, potentially eliminating the inclusion of valuable models of design, behavior change, and evaluation, which could be utilized for the development of health interventions. However, this limitation we feel is essential to understand the current state of social media health interventions.

This topic is highly relevant given the opportunities presented by social media to reach diverse audience for the purposes of promoting public and community health. This review aims to produce a piece of work that will help guide others looking to build social media health interventions toward evidence-based practices in design and evaluation, which enables the reader to also see what behaviors are being targeted and through what platforms. This review also has the potential to impact future directions of social media health interventions by illuminating the current trends of behaviors targeted by social media health interventions, revealing either an overabundance of focus in some health concerns or a gap in others. This may lay the groundwork for future research to take a closer look at the particulars of research in behavior-specific interventions.

## Abbreviations

PRISMA: Preferred Reporting Items for Systematic Reviews and Meta-Analyses
PRISMA-ScR: Preferred Reporting Items for Systematic Reviews and Meta-Analyses extension for Scoping Reviews
